# Hsa-miR-181a-5p, hsa-miR-182-5p, and hsa-miR-26a-5p as potential biomarkers for *BCR-ABL1* among adult chronic myeloid leukemia treated with tyrosine kinase inhibitors at the molecular response

**DOI:** 10.1186/s12885-022-09396-5

**Published:** 2022-03-26

**Authors:** Aliza Mohd Yacob, Nor Asiah Muhamad, Kian Meng Chang, Hamidah Akmal Hisham, Yuslina Mat Yusoff, Latifah Ibrahim

**Affiliations:** 1 Haematology Unit, Cancer Research Centre, Institute for Medical Research, National Institutes of Health, Ministry of Health, U13/52 Setia Alam, 40170 Shah Alam, Selangor Malaysia; 2 Sector for Evidence Based Healthcare, National Institutes of Health, Ministry of Health, Shah Alam, Selangor, Malaysia; 3 Haematology Department, Hospital Ampang, Ministry of Health, Ampang, Selangor Malaysia; 4 Molecular Pathology Unit, Cancer Research Centre, Institute for Medical Research, National Institutes of Health, Ministry of Health, Shah Alam, Selangor, Malaysia; 5 Special Resource Centre, Institute for Medical Research, National Institutes of Health, Ministry of Health, Shah Alam, Selangor, Malaysia

**Keywords:** Hsa-miR-181a-5p, Hsa-miR-182-5p, Hsa-miR-26a-5p, Chronic myeloid leukemia, Tyrosine kinase inhibitors, Next generation sequencing

## Abstract

**Background:**

Tyrosine kinase inhibitors (TKIs) as first-line therapy for Chronic Myeloid Leukemia (CML) show a high success rate. However, a low number of patients with long-term treatment-free remission (TFR) were observed. Molecular relapse after imatinib discontinuation occurred at 50% at 24 months, with 80% occurrence within the first 6 months. One of the reasons for relapse is untimely TKIs discontinuation caused by large errors from estimates at very low-level or undetectable disease, thus warranting new biomarkers for CML.

**Methods:**

Next Generation Sequencing (NGS) was used to identify microRNAs (miRNAs) at the molecular response in CML adult patients receiving TKIs treatment. A total of 86 samples were collected, 30 from CML patients responsive and 28 from non-responsive to imatinib therapy, and 28 from blood donors. NGS was conducted whereby 18 miRNAs were selected and validated by real-time RT-qPCR in triplicate.

**Results:**

Hsa-miR-181a-5p was expressed significantly (*p*-value< 0.05) with 2.14 and 2.33-fold down-regulation in both patient groups, respectively meanwhile hsa-miR-182-5p and hsa-miR-26a-5p were significant only in the non-responsive group with 2.08 and 2.39 fold up-regulation. The down-regulation was consistent with decreased amounts of *BCR-ABL1* in patients taking TKIs regardless of molecular responses. The up-regulation was consistent with the substantial presence of *BCR-ABL1* in CML patients treated with TKIs at the molecular response.

**Conclusions:**

Therefore, these miRNAs have potential as new therapeutic biomarkers for *BCR-ABL1* status in adult CML patients treated with TKIs at molecular responses. These could improve current approaches and require further analysis to look for targets of these miRNAs in CML.

## Background

CML occurs mainly in adults with Philadelphia chromosome (Ph) present in most patients [1] as a result of a reciprocal translocation between chromosome 9 and 22 that gives rise to *BCR-ABL1* transcripts, t(9;22)(q34.1;q11.2) [2, 3]. There are 3 phases of CML: chronic, accelerated, and blast crisis; CML is treated using Tyrosine Kinase Inhibitors (TKIs). Imatinib (Glivec®, Novartis), a first-generation TKI, has been widely used since the Food and Drug Administration (FDA) approval in 2001, given orally (400 mg) once daily, and patients in the chronic phase usually respond well to treatment [1, 4, 5]. It is a targeted therapy whereby imatinib competes for the *BCR-ABL1* tyrosine kinase site to prevent phosphorylation and inhibit proliferation [6]. Patients treated with imatinib are usually experiencing mild or moderate adverse effects [5].

Treatment progress is measured based on hematologic, cytogenetic, and molecular responses. Complete Hematologic Response (CHR) is when clinical presentations return to normal meanwhile, Complete Cytogenetic Response (CCyR) is when Ph is not detected. A molecular response is the major treatment endpoint with the optimal molecular response at *BCR-ABL1* transcript level ≤ 10% by 3 months, ≤1% by 6 months is similar to CCyR and ≤ 0.1% by 12 months, known as Major Molecular Response (MMR). Beyond optimal response is known as warning or failure whereby each indicates treatment continuation, carefully considering or changing according to the patient’s conditions. Patients non-responsive or intolerant to imatinib will be administered second and third-generation (2G or 3G) TKIs as necessary, whereby nilotinib, dasatinib, and bosutinib, 2GTKIs are now choices of front-line treatment. These enable patients to not only achieve MMR faster but also much deeper molecular responses than imatinib. However, 2G and 3G TKIs showed harsher side-effects and clinical constraints without improved overall survival [4].

The *BCR-ABL1* transcripts level quantification is by reverse transcription-quantitative polymerase chain reaction (RT-qPCR). It is a ratio of *BCR-ABL1* transcripts to *ABL1* transcripts or another internationally accepted control transcript regarding the International Randomized Study of Interferon and STI571 (IRIS) trial. It is referred to as the International Scale (IS). The IRIS standardized baseline is considered as 100% and below this or 1, 0.1, 0.01% (MR^4^), 0.0032% (MR^4.5^) and 0.001% (MR^5^) correspond to 2, 3, 4, 4.5, and 5-log reduction from the IRIS baseline respectively. MR^4^, MR^4.5^, and MR^5^ are known as deep molecular responses (DMR) [6]. A conversion factor converts laboratory ratio to an IS ratio and is determined through sample exchange with an established reference laboratory or calibrated to WHO International Genetic Reference Panel [7].

Patients who achieved DMR (MR^4^, MR^4.5^) have a quality of life near the general population; enable cessation studies in suitable patients. It is promising as losing MMR is uncommon after a year in treatment-free remission (TFR) otherwise indicates failure. Based on shared decision making, requirements for TKI discontinuation include first chronic phase, motivated patient, no prior treatment failure, typical e13a2 or e14a2 *BCR-ABL1* transcripts, more than 5 years on TKI, and achieved DMR for more than 2 or 3 years for MR^4.5^ and MR^4^ respectively [4].

DMR of 4.5-log reduction or lower is preferable for discontinuation of imatinib by controlled studies due to fewer events observed and better clinical outcomes [8–10]. These low-level estimates are measured by using RT-qPCR which gives substantial errors, particularly from Poisson variation, with a limit of detection (LoD) of 10^− 4^, 10^–4.5^, and below [11–13]. These lead to disagreements in reporting as recommended by EUTOS, particularly in assigning targets to 3 for less than 3 copies [11, 12, 14]. Among suggestions for reporting of the result were to include confidence limit [12], average targets or as an individual result for undetectable replicate [11], and LoD was proposed [15]. In most centres, the routine LoD for RT-qPCR is MR^4.5^ [16]. All these have compromised the consistency of DMR which could hinder timely TKI discontinuation; therefore, a new therapeutic biomarker is necessary.

MicroRNAs (miRNAs) are small non-protein-coding, endogenous RNAs around 22 nucleotides long and function as regulators in gene expression. The expression could be down-regulated or up-regulated. Mature miRNAs are incorporated in the RNA-induced silencing complex and annealed to mRNA. Regulation of gene expression is by mRNA cleavage or translational repression [17]. miRNAs have been studied extensively in cancer, and studies showed miRNAs have potential as a tumour suppressor or oncogene with potential use in the diagnosis, prognosis, and treatment of cancers [18–25].

NGS is suitable for detecting low-level variants as it allows deep sequencing with high sensitivity and high sequencing coverage. There are several platforms available with unique specifications [26]. Miseq system (illumina®), an easy-to-use NGS instrument that offers short sequencing run times, long read lengths, and high data quality, is suitable for targeted resequencing. The sequencing workflow involves library preparation, cluster generation, sequencing by synthesis, and data analysis. Except for library preparation, the rest are carried out by the instrument making it a reliable platform.

In this study, we investigate miRNAs of adult CML patients, responsive and non-responsive to imatinib therapy at the molecular response. Imatinib is the front-line treatment used to treat CML, and in the event of intolerance or failure, other approved TKIs are an option as appropriate. Current treatment success is measured by the minimal presence or undetectability of *BCR-ABL1* transcripts by RT-qPCR. miRNAs from the whole blood of these patients were profiled using NGS and compared with normal control. miRNAs suitable for the study were selected and verified by real-time RT-qPCR, the known gold standard for quantifying gene expression. Therefore, this study aimed to identify miRNAs that have therapeutic potential concerning TKIs at the molecular response in CML. These miRNAs could lead to discovering conditions that favour TKI discontinuation accurately, thus making long-term TFR achievable.

## Methods

### Patients and samples

A cross-sectional study was conducted among adults aged 18 and above in Ampang Hospital and National Blood Centre (NBC) Malaysia between 2013 and 2014. Of these respondents, 30 were CML patients responsive to imatinib therapy, and 28 were CML patients non-responsive to imatinib therapy taken from Ampang Hospital, while 28 were blood donors mostly from NBC. Peripheral blood samples were collected from all the respondents. A standardized data collection form was used to collect information from the respondents. The collection form has three domains that were sociodemographic information, treatment history with imatinib, and treatment status responsive or non-responsive. Responsive is defined as a CML patient assessed and confirmed by the clinician through clinical diagnostics and evaluations, receiving imatinib not less than 18 months, the latest *BCR-ABL1*^IS^ ratio was 0.1% or less and *ABL1* control gene equalled 10,000 copies or more. Non-responsive, defined as any of the above criteria that are not met and treated with imatinib, other TKIs, or other drugs as necessary. Control, defined as normal people taken from healthy donors, mostly was from the NBC who came to donate blood. The lab used a conversion factor of 0.81 to convert the *BCR-ABL1* ratio to the IS ratio. Blood samples and clinical data were collected from the clinic and sent to the research laboratory.

### Preparation of samples

Peripheral blood (2.5 ml) was collected from each participant using BD® Vacutainer Safety-Lok™ Blood Collection Set and drawn into PAXgene® Blood RNA tube (PreAnalytiX). Purification of total RNA including miRNA was conducted using; PAXgene® Blood miRNA Kit (PreAnalytiX) with silica-membrane technology. The quantity of total RNA was measured by NanoDrop® ND-1000 spectrophotometer. Meanwhile, the quality of total RNA and small RNA were analysed by the 2100 Bioanalyzer (Agilent) using Eukaryote Total RNA Nano assay (Agilent) for RNA Integrity Number (RIN) and Small RNA assay (Agilent) for the amount (%) of miRNA.

### Next generation sequencing

Total RNA of a CML patient responsive to imatinib therapy with RIN 7.7, a CML patient non-responsive to imatinib therapy with RIN 7.4, and a normal control with RIN 8.1 were selected for NGS. TruSeq Small RNA kit (illumina®); was used to prepare libraries for subsequent cluster generation. A library was generated from each sample and labelled using a unique index sequence each. The kit contained adapters designed to directly and specifically ligate to a 5′-phosphate and a 3′-hydroxyl group of mature miRNAs. The ligated small RNA was reverse transcribed and amplified with a unique index per tube to create cDNA constructs according to the protocol (Catalogue # RS-930-1012, Part # 15004197 Rev. C, March 2011). The cycling conditions for PCR amplification were 30 s at 98 °C, 15 cycles of 10 s at 98 °C, 30 s at 60 °C, and 15 s at 72 °C, followed by 10 min at 72 °C and hold at 4 °C. Each sample was analysed using High Sensitivity DNA assay (Agilent) and obtained around 150 bp band and peak each at a 100x dilution.

The cDNA constructs (library) were purified using 6% Novex TBE PAGE Gel, 1.0 mm (Invitrogen). Libraries with unique indexes were pooled, added Novex DNA Loading Dye (Invitrogen), and loaded into 2 wells (25 μl each). The gel ran for 60 min at 145 V in the XCell *SureLock*™ Mini-Cell electrophoresis unit (Invitrogen™) containing 1X TBE buffer. Upon completion, the gel was removed and stained with 0.5 mg/ml ethidium bromide solution for 2 to 3 min. It was viewed under UV light, and bands between 160 and 145 bp were cut out using a razor blade. The excised gel fragment was passed through a 0.5 ml Gel Breaker tube by centrifugation, added 300 μl of Ultra-Pure water, passed through a 5 μm Filter tube by centrifugation, and concentrated by ethanol precipitation. The pellet was then resuspended in 10 μl 10 mM Tris-HCl, pH 8.5, and kept at − 15^0^ to -25 °C. The purified sample library (different indexes) was validated using Agilent’s DNA 1000 assay (obtained a single sample peak around 150 bp) and High Sensitivity DNA assay (diluted 100x and obtained 152 bp peak, 1500 pmol/l) measured using the 2100 Bioanalyzer (Agilent).

The purified sample library was normalized to a 4 nM library using 10 mM Tris-Cl pH 8.5 with 0.1% tween 20, denatured to a 20 pM library in 1 mM NaOH using 0.2 N NaOH and pre-chilled HT1, and diluted to 13 pM using pre-chilled HT1. These were prepared according to Preparing Libraries for Sequencing on the Miseq® guide: Part # 15039740 Rev.D October 2013 (illumina®). The supplied 10 nM PhiX library was diluted to 4 nM using 10 mM Tris-Cl pH 8.5 with 0.1% tween 20, denatured to 20 pM, and further diluted to 12.5 pM. The denatured and diluted PhiX control was spiked in at least 5% to the purified sample library. The libraries were then dispensed into sample reservoir of reagent cartridge according to MiSeq® Reagent Kit v2 (50 cycles) Reagent Preparation Guide (illumina®) and ran on Miseq (illumina, USA) using Standard Flow Cell with 14 tiles imaged top and bottom for 15 M reads (approximately 5 M reads per sample). Clusters were generated and sequenced by synthesis with a single read, and then were imaged using LED and filter combinations meant for each fluorescently-labelled nucleotide. BaseSpace (illumina®) aligned, assembled, and analysed the reads. miRNAs of the responsive sample and miRNAs of the non-responsive sample were compared to miRNAs of the normal control. Eighteen miRNAs were selected mainly with regards to substantial expression level and clinical significance.

### Real-time reverse transcriptase-quantitative PCR (RT-qPCR)

The selected miRNAs were ordered as ready-to-use 96-well plate Custom miScript™ miRNA PCR Array (Qiagen). The array plate consisted of the 18 miRNAs, normalisation controls (SNORD61, SNORD95, SNORD96A, RNU6–2), reverse transcription control (miRTC), and positive PCR control (PPC) in the 24 × 4 format. cDNA was prepared using miScript® II RT kit (Qiagen) for a 20 μl reaction. The reaction consisted of 4 μl 5x miScript HiSpec Buffer, 2 μl 10x miScript Nucleic Mix, 2 μl miScript Reverse Transcriptase Mix, RNase-free water, and template RNA. A starting input of 250 ng total RNA (containing miRNA) was used as recommended, which will result in 0.5 to 1 ng cDNA per array well. The reaction was incubated for 60 min at 37 °C, followed by 5 min at 95 °C, and placed on ice. The 5x miScript HiSpec buffer facilitated the conversion of mature miRNAs into cDNA while suppressing long RNAs.

miScript® SYBR® Green PCR kit (Qiagen); was used with our 96-well plate Custom miScript™ miRNA PCR Array. A reaction mixture consisted of 1375 μl 2x QuantiTect SYBR® Green PCR Master Mix, 275 μl 10x miScript Universal Primer, 1000 μl RNase-free water, and 100 μl template cDNA. Out of this, 25 μl per well was added to the verified array plate. Then the array plate was placed in Light Cycler 480 (Roche), a real-time cycler. The cycling conditions used were; initial activation for 15 min at 95 °C, 45 cycles of; denaturation for 15 s at 94 °C followed by annealing for 30 s at 55 °C, and extension for 30 s at 70 °C. PCR amplification was followed by dissociation curve analysis performed for 10 s at 95 °C, 1 min at 65 °C, and a continuous 95 °C. The miScript PCR system used; allowed sensitive and specific detection and quantification of the mature miRNAs in triplicate in all case and control samples.

A threshold value of 1.15 was used across all PCR runs and obtained threshold cycle (C_T_) of PPC (C_T_^PPC^) value equivalent to 19 + 2 and C_T_^miRTC^- C_T_^PPC^ value less than 7, thus allowing comparison of results. These indicated high-quality RNA samples, correct cycling program run, and the correct threshold was used, and no apparent inhibition of the reverse-transcription reaction occurred, respectively. In addition, a single melting peak was exhibited in each sample representing a specific PCR product. The Light Cycler uses a crossing point with the Fit Points method while Gene Globe Data Analysis Centre (Qiagen) uses C_T,_ which is similar. Data was formatted in an Excel Spreadsheet using a provided template and uploaded into the Gene Globe Data Analysis Centre in a web browser (QIagen). Normalisation was conducted using the Global Threshold Cycle Mean of expressed miRNAs. Relative quantification was performed using ∆∆C_T_ method; whereby ∆C_T =_ C_T_^miRNA^ - C_T_ normalisation factor, while ∆∆C_T_ = ∆C_T_ (experimental sample) – ∆C_T_ (control sample). Fold change was calculated as 2^-∆∆CT^ whereby greater than 1 was reported as fold up-regulation while less than 1 as fold down-regulation. *P*-values were calculated based on the Student’s t-test of the replicate 2^(−∆CT)^ values for each miRNA. Any significant miRNA observed will be linked to TKIs received and the clinical outcomes of patients.

## Results

The majority of the respondents were Malays (51%), followed by Chinese (34%) and Indians (15%). There were more males than females in this study. The hallmark of CML (*BCR-ABL1* transcript) was present in all patients who were diagnosed with CML. In responsive patients, *BCR-ABL1* ratios showed a declining pattern with imatinib therapy. Common chromosomal abnormalities; usually seen with CML were observed such as; trisomy 8, which was seen in responsive and non-responsive patients. Loss of chromosome Y, an age-related chromosomal abnormality, was seen in Ph-negative older male patients. This study showed that the number of responsive and non-responsive patients was similar in Malays. The responsiveness is markedly higher among the Chinese. However, the numbers of Indian respondents were too small for comparison (Table 1).

**Table 1:** Demographic characteristic of CML patients responsive and non-responsive to imatinib therapy and blood donors

Characteristic	Imatinib Responsive Group (*n* = 30)	Imatinib Non-Responsive Group (*n* = 28)	Control Group (*n* = 28)
Ethnicity:			
Malays	13 (43%)	16 (57%)	15 (53%)
Chinese	12 (40%)	7 (25%)	10 (36%)
Indians	5 (17%)	5 (18%)	3 (11%)
Gender:			
Male	17 (57%)	13 (46%)	22 (79%)
Female	13 (43%)	15 (54%)	6 (21%)
Age (year):			
18 - < 45	13 (43%)	11 (39%)	22 (79%)
≥ 45	17 (57%)	17 (61%)	6 (21%)
Mean ± std. dev	48 ± 13	46 ± 14	36 ± 9

In responsive patients, 53% achieved MMR of 7 Malays, 6 Chinese, and 3 Indians. The remaining 47% achieved DMR of 6 Malays, 6 Chinese, and 2 Indians, in which 40% were with 4-log reduction (MR^4^) and 7% with 4.5-log reduction (MR^4.5^) from the IRIS baseline. Longer duration on imatinib of around 6 years (mean 71.64 ± 34.50 months) was observed in DMR patients compared to about 5.5 years (mean 66 ± 30.43 months) in MMR patients. Overall mean duration on imatinib was 5.7 years with 53% being on imatinib for more than 5 years (Table 2). In non-responsive patients, imatinib was changed to other TKIs (82%) or other treatment options (11%) according to mutations in the kinase domain and patients’ conditions and tolerability. *BCR-ABL1*^IS^ ratio was < 0.1% in 29% of patients, and the average of the latest available *BCR-ABL1*^IS^ ratio was 13.7% (Table 3).

**Table 2:** Clinical findings of CML patients responsive to imatinib therapy and a potential biomarker at the molecular response

Total No	Age (year)	Duration on imatinib (month)	IRIS molecular response (***BCR-ABL1*** ^**IS**a^)	Relative expression^**b**^ of hsa-miR-181a-5p (fold regulation) (collectively = 2.14 down-regulation)
16	25–75	26–122	MMR (≤ 0.1% or > 3-log reduction)	−5.5987 to 1.2789
12	40–71	20–131	MR^4^ (≤ 0.01% *BCR-ABL1*^IS^ or undetectable with 10,000–31,999 *ABL1* transcripts)	−8.664 to − 1.0906
2	32–54	24–50	MR^4.5^ (≤ 0.0032% *BCR-ABL1*^IS^ or undetectable with 32,000–99,999 *ABL1* transcripts)	− 2.5939 and − 1.7594
30	25-75^c^ (47.87 ± 13.23)^d^	20-131^c^ (68.63 ± 31.95)^d^		

^a^International Scale

^b^comparing to control group

^c^Range

^d^mean ± sd

**Table 3:** Clinical findings of CML patients non-responsive to imatinib therapy and potential biomarkers at the molecular response

Total No	Age (year)	Duration on TKIs (month)	IRIS molecular response (***BCR-ABL1***^**IS**a^)	Relative expression^**b**^ of hsa-miR-181a-5p (fold regulation) (collectively = 2.33 down-regulation)	Relative expression^**b**^ of hsa-miR-182-5p (fold regulation) (collectively = 2.08 up-regulation)	Relative expression^**b**^ of hsa-miR-26a-5p (fold regulation) (collectively = 2.39 up-regulation)
7	20–64	13–142	> 10%	− 41.3405 to 2.0286	−2.3320 to 9.5690	−2.9519 to 12.6264
5	43–64	47–92	≤ 10%	−6.5409 to − 1.3561	−1.5069 to 1.9297	− 1.4556 to 2.6728
6	22–49	11–58	≤ 1%	−4.3154 to −1.0566	− 1.1031 to 2.8252	− 1.5819 to 3.2229
5	21–64	35–131	≤ 0.1%	−4.4367 to − 1.3009	−1.1108 to 4.3120	− 1.3581 to 11.0684
2	36–61	29–109	MR^4^	−1.9178 and 1.0501	4.0233 and 5.8904	5.8497 and 7.4559
2	42–60	26–75	MR^4.5^	−2.3776 and 1.1333	1.1474 and 7.1027	1.1083 and 2.7102
1	64	65	undetermined	1.5916	8.1589	13.9131
28	20-64^c^ (46.04 ± 14.25)^d^	11-142^c^ (61.86 ± 36.33)^d^				

^a^International Scale

^b^comparing to control group

^c^Range

^d^mean ± sd

Profiles of miRNA expression by NGS showed a similar amount of mature miRNA was present in all samples, with 40.8% in normal control, 40.9% in responsive sample, and 40.3% in the non-responsive sample. A list of miRNAs with various expression levels was generated from each patient sample and compared to normal control. Among these, hsa-miR-181-5p expressed 0.45 and 0.51 times in responsive and non-responsive samples respectively.

**Fig. 1:**
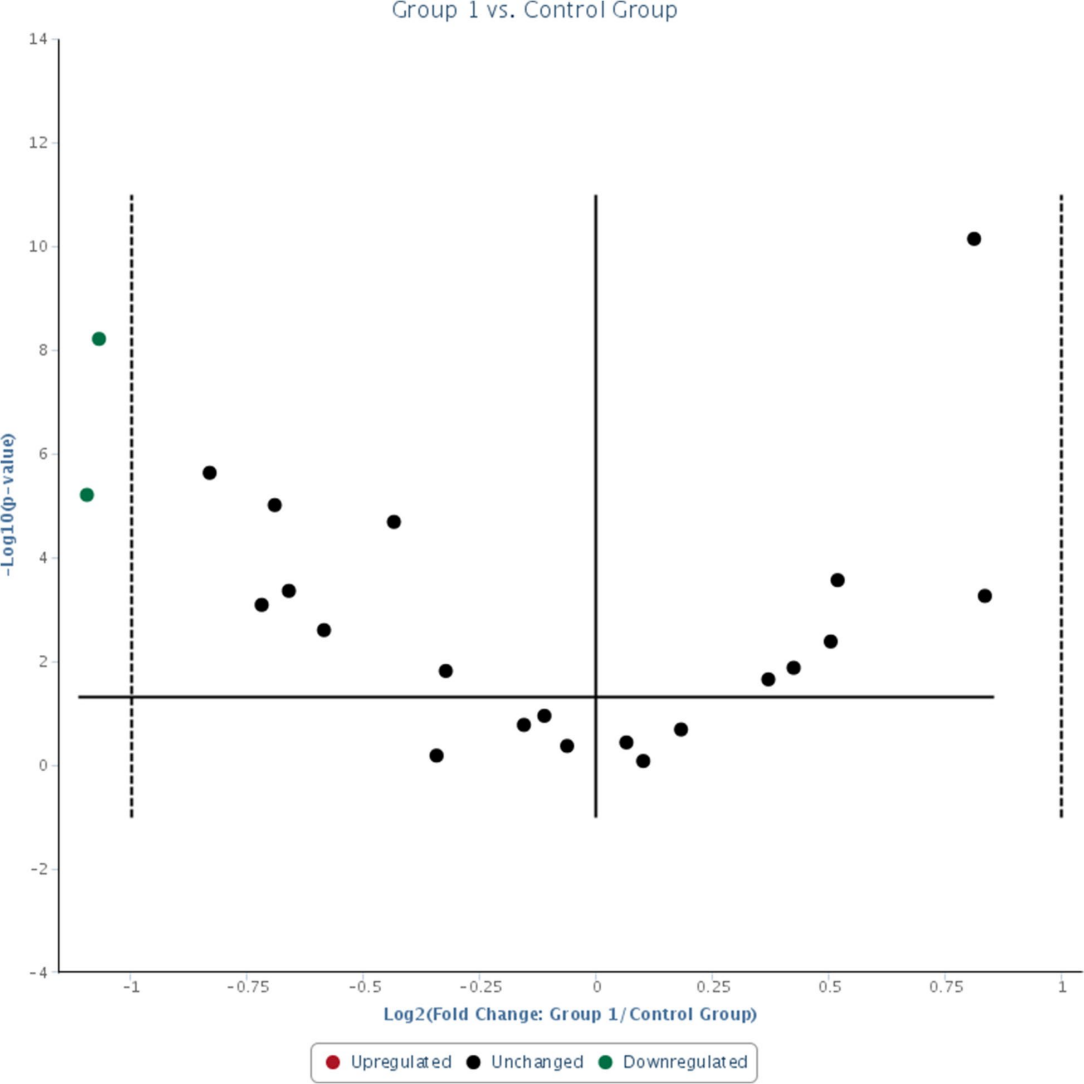
Significant down-regulation of hsa-miR-181a-5p and RNU6-2 as shown by arrows when Group 1 (CML patients responsive to imatinib therapy); was compared with the Control Group (blood donors) using custom array plate and real-time RT-qPCR. The custom array plate consisted of 18 miRNAs selected from NGS. RNU6-2 is a reference gene

**Fig. 2:**
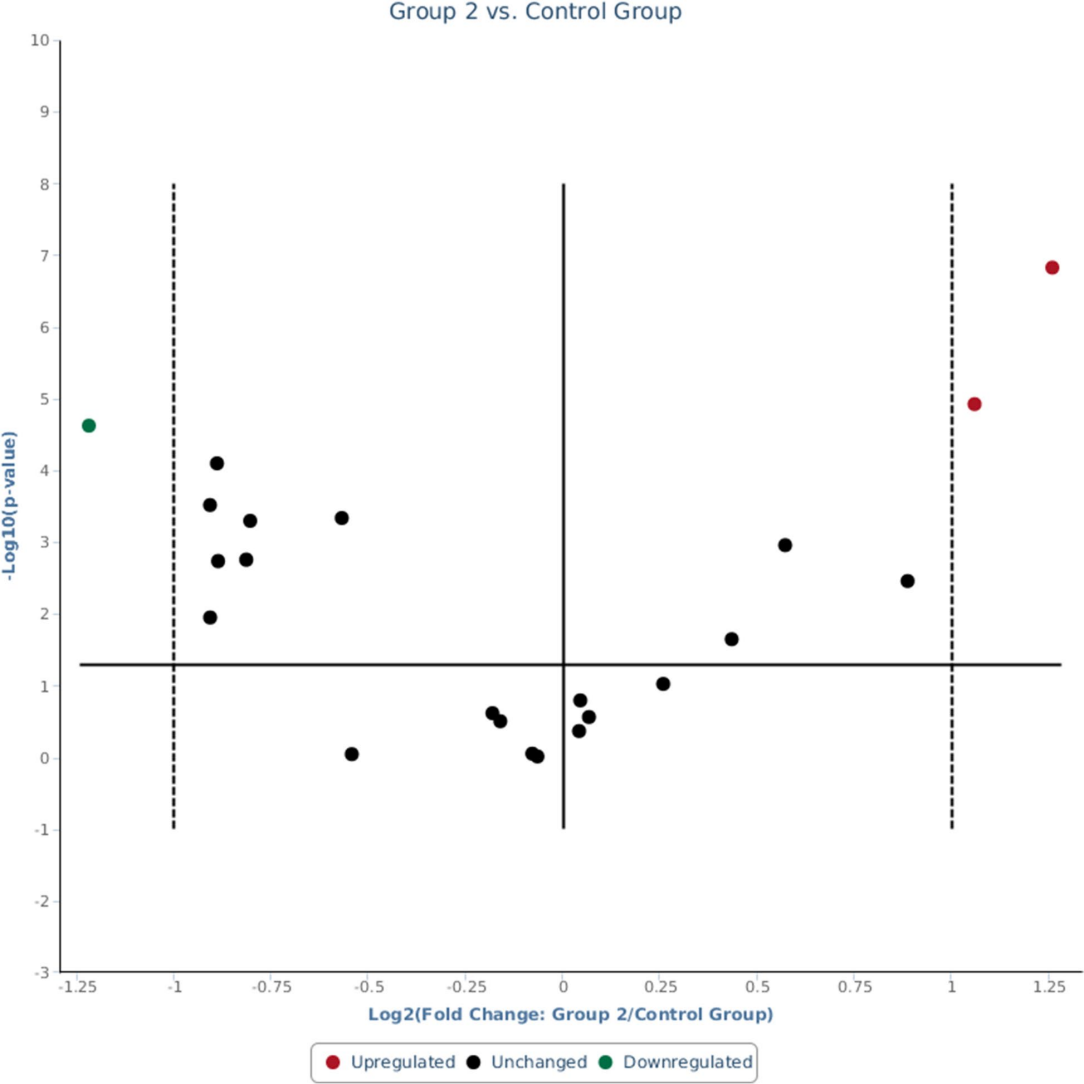
Significant down-regulation of hsa-miR-181a-5p as shown by an arrow when Group 2 (CML patients non-responsive to imatinib therapy); was compared with the Control Group (blood donors). In addition, the other two arrows showed significant up-regulation of hsa-miR-26a-5p and hsa-miR-182-5p

**Fig. 3:**
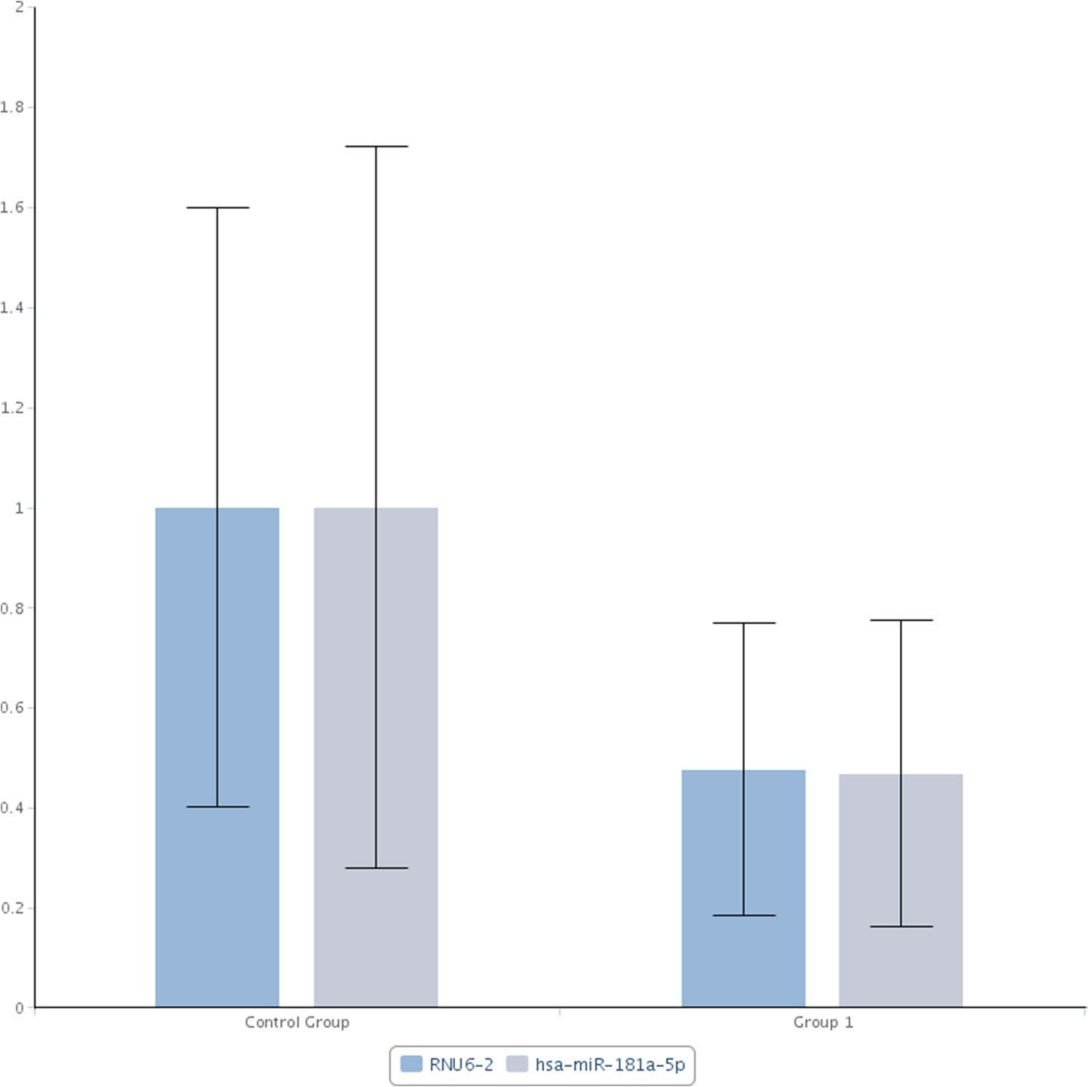
Lower expression of hsa-miR-181a-5p of 0.47 was observed in Group 1 (CML patients responsive to imatinib therapy) compared with the Control Group (blood donors) by real-time RT-qPCR (*p*-value < 0.05). RNU6–2 showed a similar expression

**Fig. 4:**
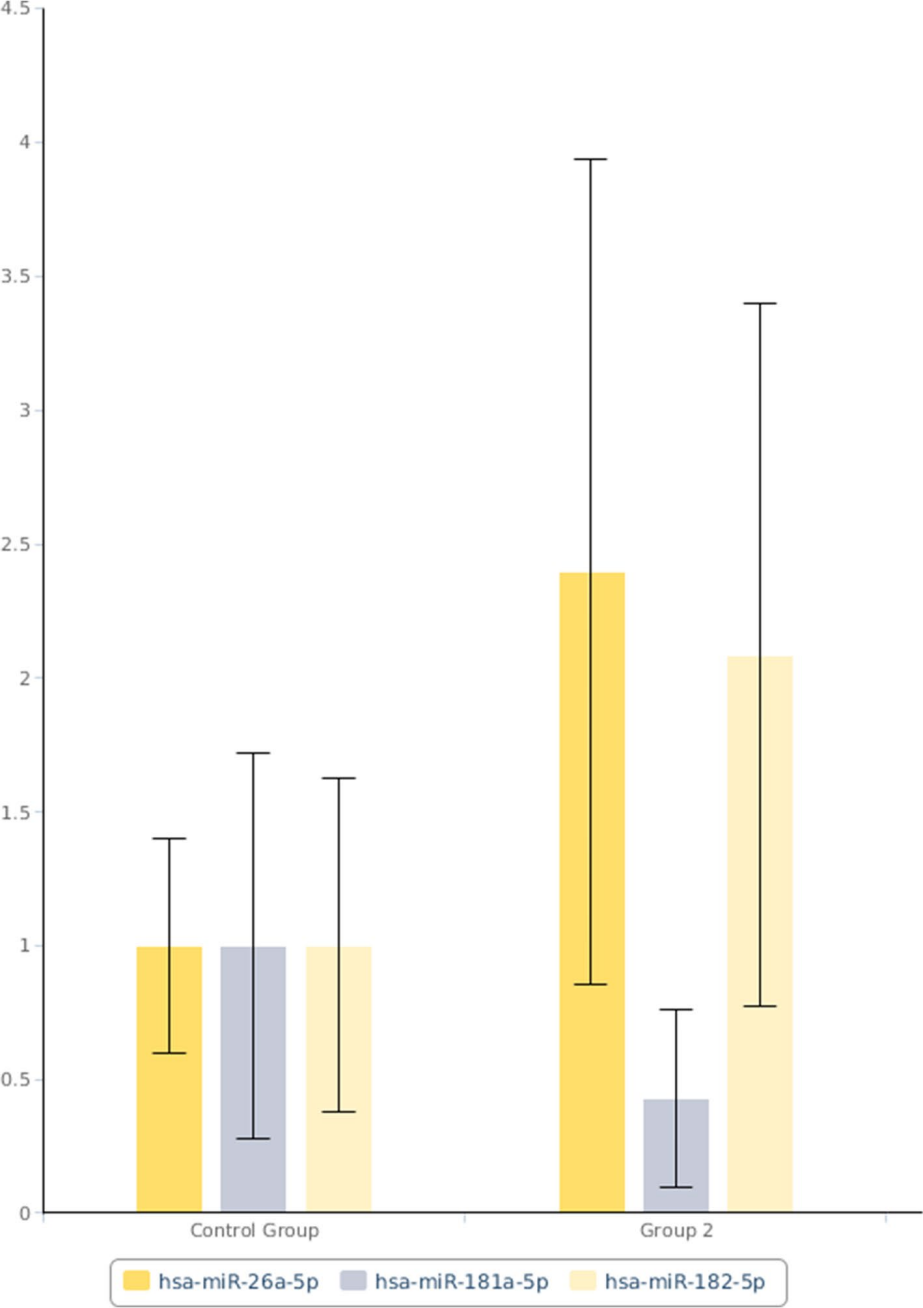
Lower expression of hsa-miR-181a-5p of 0.43 was also observed in Group 2 (CML patients non-responsive to imatinib therapy) compared with the Control Group (blood donors) by real-time RT-qPCR (*p*-value < 0.05). In addition, higher expressions were only observed from hsa-miR-182-5p and hsa-miR-26a-5p of 2.08 and 2.39, respectively, in this group

These were consistent with real-time RT-qPCR data whereby significant down-regulations of hsa-miR-181a-5p were observed in both responsive (Group 1) and non-responsive (Group 2) groups when compared to the control group (Figs. 1 and 2 respectively). Fold change of hsa-miR-181a-5p was 0.47 (95% CI = 0.36, 0.58, *p*-value = 0.000006 that was < 0.05) in responsive group (Fig. 3) and 0.43 (95% CI = 0.31, 0.55, *p*-value = 0.000024 that was < 0.05) in non-responsive group (Fig. 4) which corresponded to 2.14 and 2.33 fold down-regulation respectively compared to control group.

In addition, another 2 miRNAs were also significant but only in the non-responsive group. These were hsa-miR-182-5p with fold change of 2.08 (95% CI = 1.60, 2.57, *p*-value = 0.000012 that was < 0.05) and hsa-miR-26a-5p with fold change of 2.39 (95% CI = 1.82, 2.96, *p*-value = 0 that was < 0.05) as shown in Fig. 4 which corresponded to 2.08 and 2.39 fold up-regulation respectively when compared to the control group. The statistical analyses of real-time PCR data were as shown in Tables 4 and 5. Comparison of these biomarkers to BCR-ABL1 and IS levels were presented in Tables 2 and 3.

**Table 4:** Normalized expressions (2^(−Avg.(Delta(Ct)) of hsa-miR-181a-5p and RNU6–2 in Group 1 (CML patients responsive to imatinib therapy), and Control Group (blood donors) calculated by Gene Globe Data Analysis Centre (QIAGEN)

	Average Ct				Average Delta(Ct) (Ct(miROI) - Average Ct(normalisation factor))				2^(−Average (Delta(Ct))	
Mature ID	Control Group	Standard Deviation	Group 1	Standard Deviation	Control Group	Standard Deviation	Group 1	Standard Deviation	Control Group	Group 1
hsa-miR-181a-5p	24.99	0.804605	25.86	0.764923	5.146885	0.734814	6.241981	0.588011	0.028225	0.013212
RNU6–2	20.06	0.828910	20.90	0.754208	0.212599	0.610294	1.281648	0.640244	0.862981	0.411325

**Table 5:** Normalized miRNA expression (2^(−Avg.(Delta(Ct)) in Group 2 (CML patients non-responsive to imatinib therapy) and Control Group (blood donors) of significant miRNAs calculated by Gene Globe Data Analysis Centre (QIAGEN)

	Average Ct				Average Delta(Ct) (Ct(miROI) - Average Ct(normalisation factor))				2^(−Average(Delta(Ct))	
Mature ID	Control Group	Standard Deviation	Group 2	Standard Deviation	Control Group	Standard Deviation	Group 2	Standard Deviation	Control Group	Group 2
hsa-miR-181a-5p	24.99	0.804605	26.01	1.340582	5.146885	0.734814	6.366369	0.836165	0.028225	0.012121
hsa-miR-182-5p	23.46	0.811293	22.21	1.120019	3.621528	0.636088	2.563155	0.647466	0.081248	0.169205
hsa-miR-26a-5p	21.11	0.829467	19.65	1.452098	1.264028	0.409326	0.005655	0.833271	0.416380	0.996088

In the responsive group, a reference gene, RNU6–2, a small non-coding RNA, was also significant with fold change of 0.48 (95% CI = 0.37, 0.58, *p*-value = 0 that was < 0.05) corresponded to 2.10 fold down-regulation when compared to the control group (Fig. 3).

## Discussion

We looked at our CML patients concerning imatinib therapy, a front-line treatment for CML in our government hospitals; because of treatment discontinuation for optimal CML responders as suggested by the European LeukemiaNet (ELN) 2013 and also ELN 2020. Data showed that half of our responsive patients received imatinib therapy for more than 5 years with a mean age of 48 years old, an average of 5.5 years for patients with MMR, and 6 years for DMR. These indicated on average, MMR achieved by 18 months extended survival for at least 4 years, and DMR is achievable with a longer time on therapy in our CML patients. DMR was observed in almost half of the responsive patients, and if consistent, these patients can be considered for imatinib discontinuation studies, as minimum requirements are at least 24 months of DMR [4], and previously 12 months of DMR [27]. DMR was reported suitable for imatinib discontinuation as it has survival benefits and lesser events [8]. However, determining the right time for imatinib discontinuation is critical. Long-term TFR is still low, with relapse after discontinuation being considerably high in remission patients. The weighted mean molecular relapse rate of CML was 42%, and mean molecular remission was more than 37 months before discontinuation of imatinib. A shorter duration of imatinib therapy and a shorter time with an undetectable level of *BCR-ABL1* transcripts were reasons for relapse [28]. Therefore sufficient time on imatinib and consistency in DMR is crucial before imatinib discontinuation.

Management of CML has remarkably improved over the years with a high success rate for treatment using TKIs in chronic patients. This is assisted by new analysis techniques complementing existing ones for comprehensive detection and monitoring of CML. RT-qPCR, a sensitive technique has become a gold standard in the molecular response and is used to analyse treatment response, especially from TKIs as the first line. Since RT-qPCR has limitations at very low-levels thus new biomarkers are needed not only to assist analysis but also to understand better molecular and cellular interactions and associations to prolong TFR.

Numerous novel findings; were reported from CML studies using NGS**,** a new technology with relatively high sensitivity and high coverage. In CML patients treated with imatinib, NGS has enabled identification of novel *BCR-ABL1* fusions gene comprising *BCR* intron 14 and *ABL1* intron 2 breakpoints, giving partial deletion of SH3 domain [29]. Meanwhile, with whole-genome sequencing, NGS has enabled identification of e13a2-like *BCR-ABL1* fusion comprising broken *BCR* exon 13 and 9 *ABL1* intron 1 nucleotides forming a novel chimeric exon [30]. In our study, NGS has assisted in identifying miRNAs at the molecular response in local CML patients responsive and non-responsive to imatinib therapy. Validation by custom array real-time RT-qPCR has identified hsa-miR 181-5p, hsa-miR-182-5p, and hsa-miR-26a-5p as potential new therapeutic biomarkers in response to treatment using TKIs in CML. Hsa-miR 181-5p could be a tumour suppressor miRNA and might be involved in major or partial suppression of CML. Meanwhile, hsa-miR-182-5p and hsa-miR-26a-5p could be oncomiRs in which their expressions may indicate non-responsiveness to TKIs treatment or relapse.

Hsa-miR-181a-5p was significantly down-regulated in both the responsive and non-responsive to imatinib groups compared with the control group. The down-regulation indicated significant interactions between hsa-miR-181a-5p and TKI therapies. These showed that imatinib, nilotinib, dasatinib, and bosutinib were taken by CML patients, over time had caused inhibition to CML cell proliferation, led to apoptosis, and thus reduced the amount of *BCR-ABL1* in CML patients. The down-regulation observed in the responsive to imatinib group represented 3 to 4.5-log reduction (MMR to MR^4.5^) of *BCR-ABL1* levels from the IRIS baseline with an average of 5.7 years of intake. Similarly, down-regulation observed in the non-responsive to imatinib group represented less than 1 to 5-log reduction (approaching MMR to MR^5^) of *BCR-ABL1* levels from the IRIS baseline. These down-regulations of hsa-miR-181a-5p indicated a positive correlation to log reduction or reduced levels of *BCR-ABL1* in CML patients treated with TKIs. Therefore hsa-miR-181a-5p has the potential to be used as a therapeutic biomarker for the positive performance of TKIs shown by reduced levels of *BCR-ABL1* indicating patients were responding to TKIs treatment in CML. Hsa-miR-181a-5p could be a tumour-suppressive miRNA that prevents mRNA from coding specific protein directly or associated with CML in patients treated with TKIs.

On the other hand, hsa-miR-182-5p and hsa-miR-26a-5p were significantly up-regulated in the non-responsive to imatinib group compared with the control group. The fold up-regulation was 2.08 and 2.39, respectively, and was observed only in this group. These were patients whereby most of their latest available *BCR-ABL1*^IS^ ratios were more than 0.1% that was neither in MMR nor did DMR, with a substantial average, and only 7% [2] treated with imatinib. Fold regulation of these miRNAs in the imatinib responsive group, whereby *BCR-ABL1* in these patients was minimal or absent (MMR or DMR), was not significant compared with the control group. It indicated imatinib had reduced the amount of *BCR-ABL1* in these responsive patients to the extent not significantly different compared with healthy donors. Thus the significant up-regulation could indicate that hsa-miR-182-5p and hsa-miR-26a-5p have a positive correlation to the substantial presence of *BCR-ABL1* in CML patients treated with TKIs. Therefore, these miRNAs could function as new therapeutic biomarkers for the substantial presence of *BCR-ABL1* in CML patients treated with TKIs. Hsa-miR-182-5p and hsa-miR-26a-5p could be oncogenic miRNAs (oncomiRNAs) and might be associated with CML in patients treated with TKIs.

Studies have shown hsa-miR-181a-5p as a therapeutic biomarker, has potential use for clinical improvement in cancers. It is demonstrated in gastric cancer cells whereby hsa-miR-181a-5p has been extensively studied and reported as a potential regulator of MEG2, a tumour suppressor gene [31], regulated RASSF6, and in combination predicts poor prognosis in gastric cancer [32]. It also inhibited MTMR3 expression in AGS gastric cancer cells and was identified as novel autophagy [33]. In non-small-cell-lung cancer tissues and cell lines, hsa-miR-181a-5p was reported significantly reduced and has a potential role in tumour suppression by partially targeting Kras [34], a protein that regulates cell growth. In breast (BC) and colon cancers (CC), hsa-miR-181a-5p was reportedly down-regulated and inversely related to matrix metalloproteinase-14, which is elevated in tumours, in which, to prevent cancer metastasis in BC and CC, is by elevating hsa-miR-181a-5p [35]. In hepatocellular carcinoma: miR-181 was reported to significantly turn on the MAPK/JNK pathway, the regulator of cell proliferation, and by limiting it, would suppress the pathway [36]. Hsa-miR-181a-5p, a tumour suppressor is considered as a prognostic marker in Acute Myeloid Leukemia patients treated with intensive induction chemotherapy and autologous stem cell transplant [37].

Higher expression of hsa-miR-182-5p as observed in our study was also seen in other studies as in prostate cancer (PCa); lung squamous cell carcinoma tissues analysed from The Cancer Genome Atlas database, the Gene Expression Omnibus database, and real-time qPCR [38]; and also in colorectal cancer cells using tumorigenic variant cell line MICOL-14^tum^ compared to MICOL-14^h-tert^ cells [39]. In PCa, the biphasic role of hsa-miR-182-5p was observed, with a higher expression in localized PCa and contrarily lower expression in aggressive cancers [40]. Lower expression was also observed in renal cancer whereby hsa-miR-182-5p was down-regulated in tumour tissue compared with adjacent normal tissues, and overexpression decreased tumour growth in mice, demonstrating antitumor effect [41].

Hsa-miR-26a-5p expression in our study is consistent with a bigger-sample size study than ours on CML patients indicating the expression of miR-26a is influenced by the response to TKIs used [42]. The study showed miR-26a was differentially expressed; in HL-60.*BCR-ABL* cells when treated with tyrosine kinase inhibitors when compared to HL-60 cells. miR-26a expression levels increased gradually with both dasatinib and nilotinib treatments. The increased expression levels with dasatinib and nilotinib were consistent with our findings of hsa-miR-26a expression was up-regulated in CML patients molecularly non-responsive to imatinib compared with controls whereby most patients were treated with nilotinib or dasatinib. Thus, with dasatinib and nilotinib treatments, up-regulation of hsa-miR-26a was observed in CML patients’ non-responsiveness to imatinib. However, HL-60.*BCR-ABL* cells treated with imatinib mesylate showed increased miR-26a expression at 4-h but decreased expression at 8-h. It correlates with miR-26a expression was lower in chronic phase patients (62.5% Complete Cytogenetic Response (CCyR) and 37.5% imatinib-resistant) when compared with healthy individuals. miR-26a expression was also lower in imatinib-resistant patients when compared to patients who achieved CCyR (< 1% *BCR-ABL1*) [43]. Hsa-miR-26a-5p expression in our study was not significant in CML patients molecularly responsive to imatinib compared with controls as these were patients either in MMR or DMR (≤ 0.1% to undetectable *BCR-ABL1*). These showed that with imatinib, expressions of hsa-miR-26a were decreasing from responsiveness towards resistance. Thus these expressions indicated that imatinib, nilotinib, and dasatinib had altered *BCR-ABL* kinase activity differently.

The main limitation of this study was the low number of patient samples collected, which was due to only a small number of patients fulfilling all the required criteria within the 2-year duration. Hence, a longer time of sample collection is appropriate. In addition, our custom plate of 4 × 24 introduced variations between plates for replicates on different array plates. It made determining a suitable threshold challenging. Instead, using an array plate of 3 × 32 for triplicate is recommended.

These studies showed that hsa-miR-181a-5p, hsa-miR-182-5p, and hsa-miR-26a-5p are promising therapeutic biomarkers and can be manipulated to improve cancer treatment. In this study, only 18 miRNAs from NGS profiles were validated using real-time RT-qPCR; thus the significant miRNAs observed could be solely or partially involved in the decreasing and or increasing of *BCR-ABL1* in CML patients. Further studies are needed to determine their targets and the effect of involvement in CML. Knowing their interactions and targets in CML will give their net effects that allow manipulation by inhibiting oncomiRs or stimulating tumour suppressor miRNAs. These will give new values for TKIs responses which could assist in determining the accurate time for TKIs discontinuation and better prediction of relapse in CML. Timely discontinuation of TKIs in CML remission patients is crucial for long-term TFR. Prolong TFR would subsequently allow CML patients to achieve a good quality of life as healthy people.

## Conclusions

Hsa-miR-181a-5p has the potential as a therapeutic biomarker for *BCR-ABL1* at below current LoD in CML adult patients treated with TKIs at the molecular response and could be a tumour-suppressive miRNA. Meanwhile, hsa-miR-182-5p and hsa-miR-26a-5p have potential as therapeutic biomarkers for the substantial presence of *BCR-ABL1* in CML adults and could be oncomiRNAs.

## Data Availability

The datasets used and/or analysed during the current study are available from the corresponding author on reasonable request.

## Abbreviations

TKI: Tyrosine Kinase Inhibitor
CML: Chronic Myeloid Leukemia
TFR: Treatment free remission
NGS: Next Generation Sequencing
RT-qPCR: Reverse transcriptase-quantitative PCR
miRNA: MicroRNA
Ph: Philadelphia chromosome
IRIS: International Randomized Study of Interferon and STI571
IS: International Scale
DMR: Deep Molecular Response
CCyR: Complete Cytogenetic Response
LoD: Limit of Detection
RIN: RNA Integrity Number
PPC: Positive PCR Control
miRTC: Reverse Transcription Control
C_T_: Threshold Cycle
ELN: European LeukemiaNet
BC: Breast Cancer
CC: Colon Cancer
PCa: Prostate Cancer

## References

[1] Alikian M, Gale RP, Apperley JF, Foroni L (2017). Molecular techniques for the personalised management of patients with chronic myeloid leukaemia. Biomol Detect Quantif.

[2] Heisterkamp N, Stam K, Groffen J, de Klein A, Grosveld G (1985). Structural organization of the bcr gene and its role in the Ph’ translocation. Nature..

[3] Canaani E, Steiner-Saltz D, Aghai E, Gale RP, Berrebi A, Januszewicz E (1984). Altered transcription of an oncogene in chronic myeloid Leukaemia. Lancet..

[4] Hocchaus A (2020). European LeukemiaNet 2020 recommendations for treating chronic myeloid leukemia. Leukemia..

[5] Pretel-Irazabal M, Tuneu-Valls A, Ormaechea-Pérez N (2014). Adverse skin effects of Imatinib, a tyrosine kinase inhibitor. Actas Dermosifiliográficas.

[6] Druker BJ, Lydon NB (2000). Lessons learned from the development of an abl tyrosine kinase inhibitor for chronic myelogenous leukemia. J Clin Invest.

[7] Cross NCP, White HE, Colomer D, Ehrencrona H, Foroni L, Gottardi E (2015). Laboratory recommendations for scoring deep molecular responses following treatment for chronic myeloid leukemia. Leukemia..

[8] Kaygusuz Atagunduz I, Toptas T, Deniz R, Kara O, Eser A, Sezgin A (2017). Effects of deeper molecular responses on outcomes in chronic myeloid leukemia patients in chronic phase treated with Imatinib Mesylate. Clin Lymphoma Myeloma Leuk.

[9] Etienne G, Dulucq S, Nicolini F-E, Morisset S, Fort M-P, Schmitt A (2014). Achieving deeper molecular response is associated with a better clinical outcome in chronic myeloid leukemia patients on imatinib front-line therapy. Haematologica..

[10] Rousselot P, Charbonnier A, Cony-Makhoul P, Agape P, Nicolini FE, Varet B (2014). Loss of major molecular response as a trigger for restarting tyrosine kinase inhibitor therapy in patients with chronic-phase chronic myelogenous leukemia who have stopped imatinib after durable undetectable disease. J Clin Oncol.

[11] Young SS, Tucker T, Bosdet I, Karsan A (2016). Estimating deep molecular responses in chronic myelogenous leukemia: a Bayesian approach. Leukemia..

[12] Morley AA (2016). Reporting results for deep molecular responses in chronic myeloid leukemia. Leukemia..

[13] Latham S, Bartley PA, Budgen B, Ross DM, Hughes E, Branford S (2016). *BCR-ABL1* expression, RT-qPCR and treatment decisions in chronic myeloid leukaemia. J Clin Pathol.

[14] Ip H-W, So C-C (2015). Deep molecular response in chronic myelogenous leukemia: ensuring accuracy and consistency. Leukemia..

[15] Cross NCP, Müller MC, Hochhaus A (2015). Response to ho-wan Ip and chi-Chiu. Leukemia..

[16] Cross NC, Hochhaus A (2016). Deep molecular response in chronic myeloid leukemia. Leukemia..

[17] Bartel DP (2004). MicroRNAs. Cell.

[18] Ma L, Reinhardt F, Pan E, Soutschek J, Bhat B, Marcusson EG (2010). Therapeutic silencing of miR-10b inhibits metastasis in a mouse mammary tumor model. Nat Biotechnol.

[19] Jang E, Kim E, Son H-Y, Lim E-K, Lee H, Choi Y (2016). Nanovesicle-mediated systemic delivery of microRNA-34a for CD44 overexpressing gastric cancer stem cell therapy. Biomaterials..

[20] Dong J, Liu Y, Liao W, Liu R, Shi P, Wang L (2016). miRNA-223 is a potential diagnostic and prognostic marker for osteosarcoma. J Bone Oncol.

[21] Chen S, Sun K-X, Liu B-L, Zong Z-H, Zhao Y. MicroRNA-505 functions as a tumor suppressor in endometrial cancer by targeting TGF-$α$. Mol Cancer. 2016;15(1):11. 10.1186/s12943-016-0496-4 PMC4736705 26832151

[22] Wei Z, Liu Y, Wang Y, Zhang Y, Luo Q, Man X (2016). Downregulation of Foxo3 and TRIM31 by miR-551b in side population promotes cell proliferation, invasion, and drug resistance of ovarian cancer. Med Oncol.

[23] Sato T, Shiba-Ishii A, Kim Y, Dai T, Husni RE, Hong J (2017). miR-3941: a novel microRNA that controls IGBP1 expression and is associated with malignant progression of lung adenocarcinoma. Cancer Sci.

[24] El Fatimy R, Subramanian S, Uhlmann EJ, Krichevsky AM (2017). Genome editing reveals Glioblastoma addiction to MicroRNA-10b. Mol Ther.

[25] Bandi V, Baluchamy S (2017). miR-181a-2 downregulates the E3 ubiquitin ligase CUL4A transcript and promotes cell proliferation. Med Oncol.

[26] Quail M, Smith ME, Coupland P, Otto TD, Harris SR, Connor TR (2012). A tale of three next generation sequencing platforms: comparison of ion torrent, pacific biosciences and illumina MiSeq sequencers. BMC Genomics.

[27] Hughes TP, Ross DM (2016). Moving treatment-free remission into mainstream clinical practice in CML. Blood..

[28] Campiotti L, Basilio M, Guasti L, Piazza R, Gambacorti-passerini C, Maria A (2017). Imatinib discontinuation in chronic myeloid leukaemia patients with undetectable BCR-ABL transcript level : a systematic review and a meta-analysis. Eur J Cancer.

[29] Zhou F, Jin R, Hu Y, Mei H (2017). A novel BCR-ABL1 fusion gene with genetic heterogeneity indicates a good prognosis in a chronic myeloid leukemia case. Mol Cytogenet.

[30] Fu S, Hu Y, Fu Y, Chen F, Liu X, Zhang M (2016). Novel BCR-ABL1 fusion and leukemic mutations of SETBP1, PAX5, and TP53 detected by next generation sequencing in chronic myeloid leukemia. Cancer Biol Ther.

[31] Liu Z, Sun F, Hong Y, Liu Y, Fen M, Yin K (2017). MEG2 is regulated by miR-181a-5p and functions as a tumour suppressor gene to suppress the proliferation and migration of gastric cancer cells. Mol Cancer.

[32] Mi Y, Zhang D, Jiang W, Weng J, Zhou C, Huang K (2017). miR-181a-5p promotes the progression of gastric cancer via RASSF6-mediated MAPK signalling activation. Cancer Lett.

[33] Lin Y, Zhao J, Wang H, Cao J, Nie Y (2017). miR-181a modulates proliferation, migration and autophagy in AGS gastric cancer cells and downregulates MTMR3. Mol Med Rep.

[34] Ma Z, Qiu X, Wang D, Li Y, Zhang B, Yuan T (2015). MiR-181a-5p inhibits cell proliferation and migration by targeting Kras in non-small cell lung cancer A549 cells. Acta Biochim Biophys Sin.

[35] Li Y, Kuscu C, Banach A, Zhang Q, Pulkoski-Gross A, Kim D (2015). miR-181a-5p inhibits Cancer cell migration and angiogenesis via Downregulation of matrix Metalloproteinase-14. Cancer Res.

[36] Tan JYL, Habib NA, Chuah YW, Yau YH, Geifman-Shochat S, Chen WN (2015). Identification of cellular targets of MicroRNA-181a in HepG2 cells: a new approach for functional analysis of MicroRNAs. PLoS One.

[37] Seipel K, Messerli C, Wiedemann G, Bacher U, Pabst T (2020). MN1, FOXP1 and hsa-miR-181a-5p as prognostic markers in acute myeloid leukemia patients treated with intensive induction chemotherapy and autologous stem cell transplantation. Leuk Res.

[38] Luo J, Shi K, Yin S, Tang R, Chen W, Huang L (2018). Clinical value of miR-182-5p in lung squamous cell carcinoma: a study combining data from TCGA, GEO, and RT-qPCR validation. World J Surg Oncol.

[39] Perilli L, Tessarollo S, Albertoni L, Curtarello M, Pastò A, Brunetti E (2019). Silencing of miR-182 is associated with modulation of tumorigenesis through apoptosis induction in an experimental model of colorectal cancer. BMC Cancer.

[40] Baumann B, Acosta AM, Richards Z, Deaton R, Sapatynska A, Murphy A (2019). Association of High miR-182 levels with low-risk prostate Cancer. Am J Pathol.

[41] Kulkarni P, Dasgupta P, Bhat NS, Shahryari V, Shiina M, Hashimoto Y (2018). Elevated miR-182-5p associates with renal Cancer cell mitotic arrest through diminished MALAT-1 expression. Mol Cancer Res.

[42] Ferreira AF, Moura LG, Tojal I, Ambrósio L, Pinto-Simões B, Hamerschlak N (2014). ApoptomiRs expression modulated by BCR–ABL is linked to CML progression and imatinib resistance. Blood Cells Mol Dis.

[43] Ferreira AF, Moura LG, Tojal I, Ambrósio L, Pinto-Simões B, Hamerschlak N (2015). Corrigendum to “ApoptomiRs expression modulated by BCR-ABL is linked to CML progression and imatinib resistance” [Blood Cells, Mol Dis. 53, (1–2), (June–August 2014) 47–55]. Blood Cells Mol Dis.

